# Early management of adult traumatic spinal cord injury in patients with polytrauma: a consensus and clinical recommendations jointly developed by the World Society of Emergency Surgery (WSES) & the European Association of Neurosurgical Societies (EANS)

**DOI:** 10.1186/s13017-023-00525-4

**Published:** 2024-01-18

**Authors:** Edoardo Picetti, Andreas K. Demetriades, Fausto Catena, Bizhan Aarabi, Fikri M. Abu-Zidan, Oscar L. Alves, Luca Ansaloni, Rocco A. Armonda, Rafael Badenes, Miklosh Bala, Zsolt J. Balogh, Andrea Barbanera, Alessandro Bertuccio, Walter L. Biffl, Pierre Bouzat, Andras Buki, Ana Maria Castano-Leon, Davide Cerasti, Giuseppe Citerio, Federico Coccolini, Raul Coimbra, Carlo Coniglio, Francesco Costa, Federico De Iure, Bart Depreitere, Enrico Fainardi, Michael J. Fehlings, Nikolay Gabrovsky, Daniel Agustin Godoy, Peter Gruen, Deepak Gupta, Gregory W. J. Hawryluk, Raimund Helbok, Iftakher Hossain, Peter J. Hutchinson, Corrado Iaccarino, Kenji Inaba, Marcel Ivanov, Stanislav Kaprovoy, Andrew W. Kirkpatrick, Sam Klein, Angelos Kolias, Nikolay A. Konovalov, Alfonso Lagares, Laura Lippa, Angelica Loza-Gomez, Teemu M. Luoto, Andrew I. R. Maas, Andrzej Maciejczak, Ronald V. Maier, Niklas Marklund, Matthew J. Martin, Ilaria Melloni, Sergio Mendoza-Lattes, Geert Meyfroidt, Marina Munari, Lena M. Napolitano, David O. Okonkwo, Yasuhiro Otomo, Marios C. Papadopoulos, Ondra Petr, Wilco C. Peul, Aichholz K. Pudkrong, Zaffer Qasim, Frank Rasulo, Carla Reizinho, Florian Ringel, Sandro Rizoli, Elham Rostami, Andres M. Rubiano, Emanuele Russo, Aarti Sarwal, Jan M. Schwab, Franco Servadei, Deepak Sharma, Salman Sharif, Ehab Shiban, Lori Shutter, Philip F. Stahel, Fabio S. Taccone, Nicole A. Terpolilli, Claudius Thomé, Peter Toth, Parmenion P. Tsitsopoulos, Andrew Udy, Alexander R. Vaccaro, Albert J. Varon, Monica S. Vavilala, Alexander Younsi, Monika Zackova, Tommaso Zoerle, Chiara Robba

**Affiliations:** 1 Department of Anesthesia and Intensive Care, Parma University Hospital, Parma, Italy; 2 Department of Neurosurgery, Royal Infirmary Edinburgh, Edinburgh, UK; 3 Leiden University Neurosurgical Centre Holland, HMC-HAGA The Hague & LUMC Leiden, Leiden, The Netherlands; 4 Emergency and Trauma Surgery, Bufalini Hospital, Cesena, Italy; 5 Department of Neurosurgery, University of Maryland, Baltimore, MD USA; 6 The Research Office, College of Medicine and Health Sciences, United Arab Emirates University, Al-Ain, United Arab Emirates; 7 Hospital Lusíadas Porto, Centro Hospitalar de Gaia/Espinho, Porto, Portugal; 8 Department of Surgery, Pavia University Hospital, Pavia, Italy; 9 Department of Neurosurgery, Georgetown University School of Medicine and MedStar Washington Hospital Center, Washington, DC USA; 10 Department of Anesthesiology and Surgical-Trauma Intensive Care, Hospital Clínic Universitari de Valencia, University of Valencia, Valencia, Spain; 11 Department of General Surgery, Hadassah Medical Center and Faculty of Medicine, Hebrew University of Jerusalem, Jerusalem, Israel; 12 Department of Traumatology, John Hunter Hospital, Hunter Medical Research Institute and University of Newcastle, Newcastle, Australia; 13 Department of Neurosurgery, SS Antonio e Biagio e Cesare Arrigo Alessandria Hospital, Alessandria, Italy; 14 Division of Trauma/Acute Care Surgery, Scripps Clinic Medical Group, La Jolla, CA USA; 15 Universite Grenoble Alpes, CHU Grenoble Alpes, Grenoble, France; 16 School of Medical Sciences, Örebro University, Örebro, Sweden; 17 Neurosurgery Department, University Hospital “12 de Octubre”, Madrid, Spain; 18 Neuroradiology Unit, Parma University Hospital, Parma, Italy; 19 School of Medicine and Surgery, University of Milano-Bicocca, Monza, Italy; 20 Department of Neuroscience, Fondazione IRCCS San Gerardo Dei Tintori, Monza, Italy; 21 General, Emergency and Trauma Surgery Department, Pisa University Hospital, Pisa, Italy; 22 Division of Trauma and Acute Care Surgery, Riverside University Health System Medical Center, Riverside, CA USA; 23 Department of Anesthesia, Intensive Care and Prehospital Emergency, Ospedale Maggiore Carlo Alberto Pizzardi, Bologna, Italy; 24 Department of Neurosurgery, Fondazione IRCCS Istituto Neurologico C. Besta, University of Milan, Milan, Italy; 25 Department of Spine Surgery, Ospedale Maggiore Carlo Alberto Pizzardi, Bologna, Italy; 26 Department of Neurosurgery, University Hospital KU Leuven, Louvain, Belgium; 27 Neuroradiology Unit, Department of Experimental and Clinical Biomedical Sciences, University of Florence, Florence, Italy; 28 Division of Neurosurgery, Department of Surgery, University of Toronto, Krembil Research Institute, Toronto Western Hospital, Toronto, ON Canada; 29 Clinic of Neurosurgery, University Hospital Pirogov, Sofia, Bulgaria; 30 Neurointensive Care Unit, Sanatorio Pasteur, Catamarca, Argentina; 31 Department of Neurological Surgery, University of Southern California, Los Angeles, CA USA; 32 Department of Neurosurgery, Neurosciences Centre and JPN Apex Trauma Centre, All India Institute of Medical Sciences, New Delhi, India; 33 Neurological Institute, Cleveland Clinic, Akron General Hospital, Fairlawn, OH USA; 34 Department of Neurology, Johannes Kepler University Linz, Kepler University Hospital, Linz, Austria; 35 Department of Neurology, Medical University of Innsbruck, Innsbruck, Austria; 36 Neurocenter, Department of Neurosurgery, Turku University Hospital, Turku, Finland; 37 Division of Neurosurgery, Department of Clinical Neurosciences, Addenbrooke’s Hospital, University of Cambridge, Cambridge, UK; 38 Neurosurgery Unit, Department of Biomedical, Metabolic and Neural Sciences, University of Modena and Reggio Emilia, Azienda Ospedaliero-Universitaria di Modena, Modena, Italy; 39 Division of Acute Care Surgery, Department of Surgery, University of Southern California, Los Angeles, CA USA; 40 Neurosurgery Department, Royal Hallamshire Hospital, Sheffield, UK; 41 Department of Spinal and Peripheral Nerve Surgery Burdenko Neurosurgical Center, Moscow, Russia; 42 Departments of Surgery and Critical Care Medicine, Foothills Medical Centre, University of Calgary, Calgary, AB Canada; 43 Department of Neurosurgery, Jessa Hospital, Hasselt, Belgium; 44 Faculty of Medicine and Life Science, Hasselt University, Hasselt, Belgium; 45 ANAPLASI Rehabilitation Centre, Athens, Greece; 46 1St Neurosurgery Department, Henry Dunant Hospital Center, Athens, Greece; 47 Department of Neurosurgery, Ospedale Niguarda, Milan, Italy; 48 Department of Emergency Medicine, Perelman School of Medicine, University of Pennsylvania, Philadelphia, PA USA; 49 Department of Neurosurgery, Tampere University Hospital and Tampere University, Tampere, Finland; 50 Department of Neurosurgery, Antwerp University Hospital, Edegem, Belgium; 51 Department of Translational Neuroscience, Faculty of Medicine and Health Science, University of Antwerp, Antwerp, Belgium; 52 Department of Neurosurgery, St Luke Hospital, University of Rzeszow, Tarnow, Poland; 53 Department of Surgery, University of Washington, Seattle, WA USA; 54 Department of Clinical Sciences Lund, Neurosurgery, Lund University, Lund, Sweden; 55 Department of Neurosurgery, Skåne University Hospital, Lund, Sweden; 56 University of Southern California, Los Angeles, CA USA; 57 Division of Neurosurgery, Department of Neurosciences (DINOGMI), IRCCS Ospedale Policlinico San Martino, Genoa, Italy; 58 Department of Orthopaedics, Duke University, Durham, NC USA; 59 Department and Laboratory of Intensive Care Medicine, University Hospitals Leuven and KU Leuven, Louvain, Belgium; 60 Neuro-Intensive Care Unit, University Hospital of Padova, Padua, Italy; 61 Department of Surgery, University of Michigan Health System, Ann Arbor, MI USA; 62 Department of Neurological Surgery, University of Pittsburgh, Pittsburgh, PA USA; 63 National Disaster Medical Center, Tokyo, Japan; 64 Academic Neurosurgery Unit, St. George’s, University of London, London, UK; 65 Department of Neurosurgery, Medical University Innsbruck, Innsbruck, Austria; 66 Department of Anesthesiology and Pain Medicine, University of Washington, Seattle, WA USA; 67 Department of Neuroanesthesia and Neurocritical Care, Spedali Civili University Affiliated Hospital of Brescia, Brescia, Italy; 68 Departamento de Neurocirurgia, Hospital Egas Moniz, Centro Hospitalar Lisboa Ocidental, Lisbon, Portugal; 69 Department of Neurosurgery, University Hospital Mainz, Mainz, Germany; 70 Trauma Surgery Department, Hamad General Hospital, HMC, Doha, Qatar; 71 Section of Neurosurgery, Department of Medical Sciences, Uppsala University, Uppsala, Sweden; 72 Neurosciences Institute, El Bosque University, Bogotá, Colombia; 73 Anesthesia and Intensive Care Unit, AUSL Romagna, M.Bufalini Hospital, Cesena, Italy; 74 Department of Neurology, Atrium Wake Forest School of Medicine, Winston Salem, NC USA; 75 Belford Center for Spinal Cord Injury and Departments of Neurology and Neuroscience, Wexner Medical Center, The Ohio State University, Columbus, OH USA; 76 Humanitas Research Hospital-IRCCS & Humanitas University, Rozzano, Milan Italy; 77 Neuroanesthesia & Perioperative Neuroscience, University of Washington, Seattle, WA USA; 78 Department of Neurosurgery, Liaquat National Hospital, Karachi, Pakistan; 79 Department of Neurosurgery, University Hospital Augsburg, Augsburg, Germany; 80 Department of Critical Care Medicine, Neurology and Neurosurgery, University of Pittsburgh School of Medicine, Pittsburgh, PA USA; 81 Department of Surgery, Brody School of Medicine, East Carolina University, Greenville, NC USA; 82 Department of Intensive Care, Hopital Universitaire de Bruxelles (HUB), Université Libre de Bruxelles (ULB), Brussels, Belgium; 83 Department of Neurosurgery, LMU Hospital, Ludwig-Maximilian-University Munich, Munich, Germany; 84 Department of Neurosurgery, Medical School, University of Pecs, Pecs, Hungary; 85 Department of Neurosurgery, Hippokration General Hospital, Aristotle University School of Medicine, Thessaloníki, Greece; 86 Department of Intensive Care and Hyperbaric Medicine, The Alfred, Melbourne, VIC 3004 Australia; 87 Department of Orthopedic Surgery, Delaware Valley Spinal Cord Injury Center, Rothman Orthopedics, Sidney Kimmel Medical Center of Thomas Jefferson University, Philadelphia, PA USA; 88 Department of Anesthesiology, Perioperative Medicine, and Pain Management, University of Miami Miller School of Medicine/Ryder Trauma Center, Miami, FL USA; 89 Department of Neurosurgery, Heidelberg University Hospital, Heidelberg, Germany; 90 Division of Intensive Care and Neurology Unit, Montecatone Rehabilitation Institute, Imola, Italy; 91 Department of Pathophysiology and Transplantation, University of Milan, Department of Anesthesia, Critical Care and Emergency, Fondazione IRCCS Ca’ Granda Ospedale Maggiore Policlinico, Milan, Italy; 92 IRCCS Policlinico San Martino, Dipartimento di Scienze Chirurgiche Diagnostiche e Integrate, Università di Genova, Genoa, Italy

**Keywords:** Traumatic spinal cord injury, Polytrauma, Management

## Abstract

**Background:**

The early management of polytrauma patients with traumatic spinal cord injury (tSCI) is a major challenge. Sparse data is available to provide optimal care in this scenario and worldwide variability in clinical practice has been documented in recent studies.

**Methods:**

A multidisciplinary consensus panel of physicians selected for their established clinical and scientific expertise in the acute management of tSCI polytrauma patients with different specializations was established. The World Society of Emergency Surgery (WSES) and the European Association of Neurosurgical Societies (EANS) endorsed the consensus, and a modified Delphi approach was adopted.

**Results:**

A total of 17 statements were proposed and discussed. A consensus was reached generating 17 recommendations (16 strong and 1 weak).

**Conclusions:**

This consensus provides practical recommendations to support a clinician’s decision making in the management of tSCI polytrauma patients.

**Supplementary Information:**

The online version contains supplementary material available at 10.1186/s13017-023-00525-4.

## Background

Traumatic spinal cord injury (tSCI) is a devastating condition associated with high mortality and morbidity [1, 2]. Falls from height and road traffic collisions are the most frequent causes of tSCI [1, 2]. In the latter scenario, an association of multisystem trauma was observed in about 80% of accidents [3]. In tSCI, as in traumatic brain injury (TBI), both primary and secondary injuries can occur; the latter, in particular, can be further exacerbated by dangerous secondary insults (such as hypoxia and hypotension) frequently observed in unstable polytrauma patients [3, 4]. Increased organ system injuries are associated with greater intrahospital mortality in polytrauma patients with cervical SCI [5, 6]. Unfortunately, few data are available regarding the acute phase management of tSCI patients with multisystem trauma. A recent survey showed a great worldwide variability in clinical practices in this setting [7]. Moreover, as in TBI, early therapeutic choices can deeply impact the outcome and prognosis of tSCI patients.

Considering the above, the specific aim of this consensus is to provide recommendations on the early management (within 24 h from trauma) of adult tSCI patients with polytrauma.

## Methods

An international multidisciplinary consensus panel was composed, including neurosurgeons (*n* = 44), anesthesiologists/intensivists/neurointensivists (*n* = 23), acute care surgeons (*n* = 14), orthopedists (*n* = 3), neuroradiologists (*n* = 2), emergency physicians (*n* = 2) and neurologists (*n* = 1) having expertise in tSCI polytrauma care (Additional file 1: Appendix 1). The methodology used was similar to previously published consensus conferences [8, 9]. Briefly, following a non-systematic review of the literature, the steering committee (EP, CR and FC) generated a list of questions to be assessed by the panel. Two subsequent online questionnaires were administered between May and September 2023. The list of statements (17) was formulated and distributed to the panelists to allow modifications or additional statements. The analysis of voting results was performed by a non-voting experienced methodologist (CR). Statements were classified as strong recommendation, weak recommendation and no recommendation when > 85%, 75–85% and < 75% of votes were in favor, respectively. This consensus is for tSCI polytrauma patients without severe TBI. Regarding the early management of severe TBI in polytrauma patients, we refer to the 2019 World Society of Emergency Surgery (WSES) consensus conference [8]. The current consensus was endorsed jointly by the WSES and the European Association of Neurosurgical Societies (EANS).

## Results

**Fig. 1:**
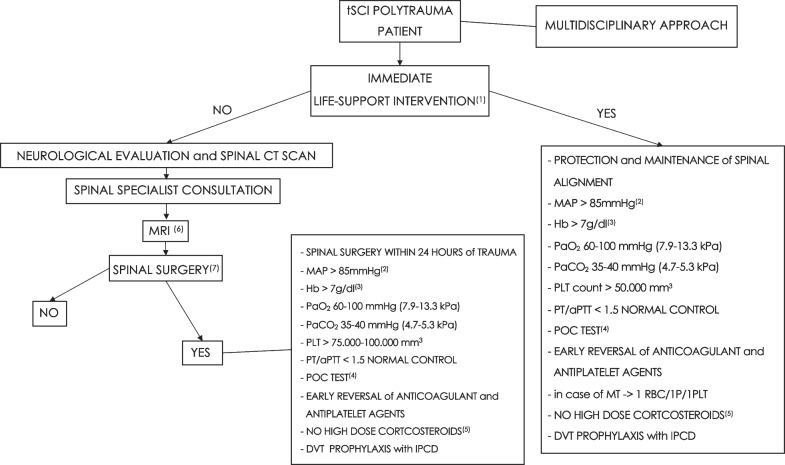
Consensus flowchart. **(1)** intubation, mechanical ventilation, hemodynamic support, extraspinal surgery/interventional radiology for bleeding control, etc. **(2)** In case of difficult intraoperative bleeding control, lower values could be tolerated for the shortest possible time. **(3)** higher values in patients "at risk" (e.g., elderly and/or with limited cardiovascular reserve because of pre-existing heart disease, etc.). **(4)** if available (e.g., TEG, ROTEM, etc.). **(5)** as utilized in NABISC I-III studies. **(6)** in order to determine the severity of spinal cord damage and aid in surgical decision making. **(7)** decompression and/or stabilization. tSCI = Traumatic spinal cord injury, CT = Computed tomography, MRI = Magnetic resonance imaging, MAP = Mean arterial pressure, Hb = Hemoglobin, PaO_2_ = Arterial partial pressure of oxygen, PaCO_2_ = Arterial partial pressure of carbon dioxide, PLT = Platelet, PT = Prothrombin time, aPTT = Activated partial thromboplastin time, POC = Point-of-care, P = Plasma, RBC = Red blood cell, MT = Massive transfusion, DVT = Deep vein thrombosis, IPCD = Intermittent pneumatic compression device

The consensus provided 17 recommendations (Table 1): 16 were strong recommendations, while 1 was a weak recommendation. The consensus algorithm is reported in Fig. 1. The consensus recommendations are listed below with the percentage of agreement.

**Table 1:** List of consensus recommendations

N	Recommendation	Level
1	We recommend that all salvageable tSCI polytrauma patients with life-threatening conditions need immediate life support interventions (e.g., intubation, mechanical ventilation, hemodynamic support, extraspinal surgery/interventional radiology for bleeding control, etc.)	Strong recommendation
2	We recommend that tSCI polytrauma patients needing extracranial interventions (e.g., surgery, interventional radiology, etc.) for life-threatening conditions require careful positioning (protection and maintenance of spine alignment) to avoid secondary insults to the injured spine	Strong recommendation
3	We recommend that tSCI polytrauma patients, without life-threatening conditions or after cardiorespiratory stabilization, need urgent neurological evaluation and imaging (i.e., spine reconstruction from a whole-body CT scan)	Strong recommendation
4	We recommend, in tSCI polytrauma patients (without life-threatening conditions or after cardiorespiratory stabilization), to perform spine MRI after spinal specialist consultation to determine the severity of spinal cord damage and aid in surgical decision making	Strong recommendation
5	We recommend, in all salvageable tSCI polytrauma patients with surgical spinal lesions, after control of life-threatening conditions, to consider an urgent intervention (decompression/spine stabilization), possibly within 24 h from trauma	Strong recommendation
6	We recommend the maintenance of a MAP > 85 mmHg during interventions for life-threatening hemorrhage or emergency spinal surgery. In case of difficult intraoperative bleeding control, lower values could be tolerated for the shortest possible time	Strong recommendation
7	We recommend that the Hb level, during interventions for life-threatening hemorrhage or emergency spinal surgery, be adjusted according to a patient's tolerance to anemia. An Hb < 7 g/dl should promptly trigger RBCTs. A higher threshold for RBCTs could be used in patients "at risk" (e.g., elderly and/or with limited cardiovascular reserve because of pre-existing heart disease, etc.)	Strong recommendation
8	We recommend the maintenance of a PaO_2_ between 60 and 100 mmHg (7.9–13.3 kPa) during interventions for life-threatening hemorrhage or emergency spinal surgery	Strong recommendation
9	We recommend the maintenance of a PaCO_2_ between 35 and 40 mmHg (4.7–5.3 kPa) during interventions for life-threatening hemorrhage or emergency spinal surgery	Strong recommendation
10	In case of interventions for life-threatening hemorrhage, we recommend the maintenance of a PLT count > 50.000/mm^3^. In the case of spinal surgery (decompression/stabilization), a higher value (75.000–100.000/mm^3^) would be advisable	Strong recommendation
11	We recommend the maintenance of a PT/aPTT value < 1.5 normal control during interventions for life-threatening hemorrhage or emergency spinal surgery	Strong recommendation
12	We recommend, if available, the utilization of POC tests (e.g., TEG, ROTEM, etc.) to assess and optimize the coagulation function during interventions for life-threatening hemorrhage or emergency spinal surgery	Weak recommendation
13	We recommend the early reversal of anticoagulant/antiplatelet agents in all salvageable tSCI polytrauma patients needing interventions for life-threatening hemorrhage or emergency spinal surgery	Strong recommendation
14	In adult patients with tSCI and polytrauma, we recommend the transfusion of RBCs/Plasma/PLTs at a ratio of 1/1/1 during massive transfusion protocol initiation. Afterward, this ratio can be modified according to laboratory values	Strong recommendation
15	We recommend against high-dose corticosteroid therapy (e.g., NASCIS I-III) in tSCI patients and polytrauma	Strong recommendation
16	We recommend DVT prophylaxis with intermittent pneumatic compression devices (if available and feasible) as soon as possible after tSCI and polytrauma	Strong recommendation
17	We recommend a strict collaboration between the different medical specialties (e.g., critical care medicine, acute care surgery, neurosurgery, neurology, emergency medicine, orthopedics, etc.) involved in the early management of tSCI patients with polytrauma	Strong recommendation

*tSCI* Traumatic spinal cord injury, *CT* Computed tomography, *MRI* Magnetic resonance imaging, *MAP* Mean arterial pressure, *Hb* Hemoglobin, *RBC* Red blood cell, *RBCT* RBC transfusion, *PLT* Platelet, *PT* Prothrombin time, *aPTT* Activated partial thromboplastin time, *POC* Point-of-care, *TEG* Thromboelastography, *ROTEM* Rotational thromboelastometry, *PaCO*_*2*_ Arterial partial pressure of carbon dioxide, *PaO*_*2*_ Arterial partial pressure of oxygen, *NASCIS* National Acute Spinal Cord Injury Study

### Recommendation 1

We recommend that all salvageable tSCI polytrauma patients with life-threatening conditions need immediate life support interventions (e.g., intubation, mechanical ventilation, hemodynamic support, extraspinal surgery/interventional radiology for bleeding control, etc.) (agreement: 98.9%, *strong* recommendation).

### Recommendation 2

We recommend that tSCI polytrauma patients needing extracranial interventions (e.g., surgery, interventional radiology, etc.) for life-threatening conditions require careful positioning (protection and maintenance of spine alignment) to avoid secondary insults to the injured spine (agreement: 100%, *strong* recommendation).

### Recommendation 3

We recommend that tSCI polytrauma patients, without life-threatening conditions or after cardiorespiratory stabilization, need urgent neurological evaluation and imaging [i.e., spine reconstruction from a whole-body computed tomography (CT) scan] (agreement: 95.5%, *strong* recommendation).

### Recommendation 4

We recommend, in tSCI polytrauma patients (without life-threatening conditions or after cardiorespiratory stabilization), to perform spine magnetic resonance imaging (MRI) after spinal specialist consultation to determine the severity of spinal cord damage and aid in surgical decision making (agreement: 95.5%, *strong* recommendation).

### Recommendation 5

We recommend, in all salvageable tSCI polytrauma patients with surgical spinal lesions, after control of life-threatening conditions, to consider an urgent intervention (decompression/spine stabilization), possibly within 24 h from trauma (agreement: 92%, *strong* recommendation).

### Recommendation 6

We recommend the maintenance of a mean arterial pressure (MAP) > 85 mmHg during interventions for life-threatening hemorrhage or emergency spinal surgery. In case of difficult intraoperative bleeding control, lower values could be tolerated for the shortest possible time (agreement: 88.6%, *strong* recommendation).

### Recommendation 7

We recommend that the hemoglobin (Hb) level, during interventions for life-threatening hemorrhage or emergency spinal surgery, be adjusted according to patient's tolerance to anemia. An Hb < 7 g/dl should promptly trigger red blood cell transfusions (RBCTs). A higher threshold for RBCTs could be used in patients "at risk" (e.g., elderly and/or with limited cardiovascular reserve because of pre-existing heart disease, etc.) (agreement: 92%, *strong* recommendation).

### Recommendation 8

We recommend the maintenance of an arterial partial pressure of oxygen (PaO_2_) between 60 and 100 mmHg (7.9–13.3 kPa) during interventions for life-threatening hemorrhage or emergency spinal surgery (agreement: 93.2%, *strong* recommendation).

### Recommendation 9

We recommend the maintenance of an arterial partial pressure of carbon dioxide (PaCO_2_) between 35 and 40 mmHg (4.7–5.3 kPa) during interventions for life-threatening hemorrhage or emergency spinal surgery (agreement: 93.2%, *strong* recommendation).

### Recommendation 10

In case of interventions for life-threatening hemorrhage, we recommend the maintenance of a platelet (PLT) count > 50.000/mm^3^. In the case of spinal surgery (decompression/stabilization), a higher value (75.000–100.000/mm^3^) would be advisable (agreement: 93.2%, *strong* recommendation).

### Recommendation 11

We recommend the maintenance of a prothrombin time (PT)/activated partial thromboplastin time (aPTT) value < 1.5 normal control during interventions for life-threatening hemorrhage or emergency spinal surgery (agreement: 92%, *strong* recommendation).

### Recommendation 12

We recommend, if available, the utilization of point-of-care (POC) tests [e.g., thromboelastography (TEG), rotational thromboelastometry (ROTEM), etc.] to assess and optimize the coagulation function during interventions for life-threatening hemorrhage or emergency spinal surgery (agreement: 84.1%, *weak* recommendation).

### Recommendation 13

We recommend the early reversal of anticoagulant/antiplatelet agents in all salvageable tSCI polytrauma patients needing interventions for life-threatening hemorrhage or emergency spinal surgery (agreement: 92%, *strong* recommendation).

### Recommendation 14

In adult patients with tSCI and polytrauma, we recommend the transfusion of RBCs/Plasma/PLTs at a ratio of 1/1/1 during massive transfusion protocol initiation. Afterward, this ratio can be modified according to laboratory values (agreement: 94.3%, *strong* recommendation).

### Recommendation 15

We recommend against high-dose corticosteroid therapy (i.e., National Acute Spinal Cord Injury Study (NASCIS) I-III [10–12]) in tSCI patients and polytrauma (agreement: 89.8%, *strong* recommendation).

### Recommendation 16

We recommend deep vein thrombosis (DVT) prophylaxis with intermittent pneumatic compression devices (if available and feasible) as soon as possible after tSCI and polytrauma (agreement: 94.3%, *strong* recommendation).

### Recommendation 17

We recommend a strict collaboration between the different medical specialties (e.g., critical care medicine, acute care surgery, neurosurgery, neurology, emergency medicine, orthopedics, etc.) involved in the early management of tSCI patients with polytrauma (agreement: 98.9%, *strong* recommendation).

## Discussion

### Cardiorespiratory management and bleeding control in tSCI polytrauma patients

Life-threatening hemorrhage is a major preventable cause of early death after trauma [13]. Bleeding control with circulating blood volume restoration is a priority in polytrauma care and the main goal of damage control strategies [14, 15]. Arterial hypotension and hypoxia are frequently observed after isolated tSCI, especially when the injury occurs at high spinal cord levels [1]. These secondary insults, associated with an unfavorable neurological outcome, may occur further in severe polytrauma patients [1, 3]. The most recent guidelines by the Congress of Neurological Surgeons (CNS) for managing tSCI patients recommend maintaining MAP between 85 and 90 mmHg for the first 7 days following an acute cervical SCI (Level III) [16]. These values are difficult to obtain in the acute phase management of bleeding polytrauma patients, also considering the worldwide increase in the utilization of permissive hypotension in the setting of damage control resuscitation [14]. Considering the above, we recommend the maintenance of a MAP > 85 mmHg during interventions for life-threatening hemorrhage or emergency spinal surgery. However, in case of difficult intraoperative bleeding control, we suggest tolerating lower values for the time strictly necessary to achieve bleeding control. Some panelists suggested individualizing an arterial pressure target, considering the spinal cord perfusion pressure (SCPP) [17]. To obtain this, it is necessary to monitor the intraspinal pressure (ISP) by surgically implanting an intradural extramedullary probe at the injury site [17]. Increased SCPP is associated with decreased spinal cord ischemia and favorable neurological outcomes [18–22]. At present, more data are necessary to spread this promising type of monitoring into daily clinical practice. Data about optimal Hb values in tSCI are lacking [16, 23]. We recommend RBCTs in case of Hb < 7 g/dl according to current guidelines [8, 24] and the tolerance to anemia; in this regard, a higher Hb threshold could be used in patients "at risk" (e.g., elderly and/or with limited cardiovascular reserve because of pre-existing heart disease, etc.). As for Hb values, data regarding optimal PaO_2_ and PaCO_2_ targets in this setting are lacking. Our recommendations are probably influenced by what is suggested in patients with acute brain damage [25]. 

### Imaging and spinal surgery timing

Whole-body CT scan can be considered a crucial standard diagnostic tool in the acute management of severely injured patients [26, 27]. CT scan is also recommended as the initial imaging modality evaluation of tSCI [28, 29]. Spine reconstruction from a whole-body CT scan can be quickly obtained, limiting the waste of time in the potentially challenging polytrauma setting [30]. Precisely, for the cervical spine, the appropriate standard is a thin slice helical CT scan from the base of the skull to at least the first thoracic vertebra (T1) with both sagittal and coronal reconstructions [30]. This examination should be undertaken as standard practice with the first CT brain scan in all head-injured patients with an altered level of consciousness [30]. The remaining thoracic and lumbar spine may be adequately imaged by sagittal and coronal reformatting of helical CT scans of the chest, abdomen and pelvis as part of a modern CT trauma series [30]. CT provides important information regarding bone pathology (e.g., fractures), disk herniations and epidural/subdural hematomas [28–30]. MRI, allowing a precise evaluation of the spinal cord and related soft tissues, is considered the imaging modality of choice for the evaluation of tSCI patients and for the decision of final neurosurgical/spinal treatment [30, 31]. Recent guidelines suggest MRI examination: (a) before surgical intervention, when feasible, to facilitate improved clinical decision making (Quality of Evidence: Very Low, Strength of Recommendation: Weak) and (b) in the acute period following SCI, before or after surgical intervention, to improve prediction of neurological outcome (Quality of Evidence: Low Strength of Recommendation: Weak) [31]. MRI requires more time from execution with respect to CT scan and can be challenging in cardiorespiratory unstable polytrauma patients. Precise MRI indications and timing in tSCI patients are not clearly defined, and in this regard, more studies are necessary. In the meantime, we recommend performing spine MRI after cardiorespiratory stabilization and spinal specialist consultation to determine the severity of spinal cord damage and aid in surgical decision making. Early decompressive surgery (performed within 24 h from trauma) seems to be associated with better neurological outcomes, highlighting the concept of “time is spine” [32–34]. A more rapid approach (within 12 h or less—“ultra-early”) was also proposed, especially in the case of incomplete spinal lesion [34]. Recent guidelines suggest early surgery (< 24 h after injury) as a treatment option for adult acute SCI patients, regardless of level (Quality of Evidence: Low. Strength of Recommendation: Weak) [35]. Some panelists suggest a wider timing in case of spinal fracture needing surgical stabilization in the absence of neural compression. The optimal timing of spinal surgery in tSCI polytrauma patients must be established and probably individualized according to clinical needs and after intracranial, hemodynamic and respiratory stabilization.

### Coagulation management

In bleeding polytrauma patients, an attempt is generally made to maintain PT/aPTT < 1.5 times the normal control and the PLT count > 50,000/mm^3^ [31]. A PLT count > 100,000/mm^3^ was recommended for patients with ongoing bleeding and/or TBI and in the case of neurosurgery [24, 36]. POC tests (i.e., TEG, ROTEM, etc.) are increasingly used to evaluate coagulation function in bleeding trauma patients [24]. These tests are useful for a rapid and precise assessment of hemostasis and to provide critical information about specific coagulation deficiencies, especially in patients taking novel oral anticoagulants (NOACs) and in the evaluation of PLTs dysfunction induced by trauma and/or drugs [24]. Several published guidelines about the reversal of anticoagulant/antiplatelets in different settings are available; these, especially regarding antiplatelet drug reversal, provide conflicting indications [24, 37–39]. To our knowledge, no specific guidelines about coagulation management in tSCI patients have been published until now. More studies are needed to provide robust recommendations in this setting. Meanwhile, we recommend the early reversal of anticoagulant/antiplatelet agents in tSCI polytrauma patients needing interventions for life-threatening hemorrhage or emergency spinal surgery. Massive transfusion is frequently utilized in bleeding trauma patients [14, 40]. The pragmatic randomized optimal platelet and plasma ratios (PROPPR) study, involving 680 trauma patients with major bleeding, was performed to determine the safety and the effectiveness of a transfusion strategy involving plasma, PLTs and RBCs in a 1:1:1 ratio compared with a 1:1:2 ratio. None of the studied strategies resulted in significant differences in mortality. However, patients in the 1:1:1 group achieved hemostasis with fewer deaths due to exsanguination within the first 24 hours [41]. Some of our panelists suggest, as an alternative to this strategy, the utilization of whole blood transfusion as indicated in a recently published clinical protocol for damage control resuscitation by the American Association for the Surgery of Trauma (AAST) and the American College of Surgeons Committee on Trauma (ACS-COT) [14]. No data are available regarding trauma coagulopathy and the progression of tSCI. Therefore, we recommend initiating a transfusion protocol of RBCs/plasma/PLTs at a ratio of 1:1:1. This ratio may be modified afterward according to laboratory values. Patients with SCI are at increased risk of developing venous thromboembolism after trauma [42]. Early initiation of mechanical thromboprophylaxis with intermittent pneumatic compression was recommended in immobile trauma patients with elevated bleeding risk [24, 43]. In this regard, we recommend DVT prophylaxis with intermittent pneumatic compression devices (if available and feasible) as soon as possible after tSCI and polytrauma. The initiation of pharmacologic DVT prophylaxis should be considered within 48 h of injury or spine surgery, as suggested by recent published guidelines [43]. 

### Corticosteroid therapy

The utilization of corticosteroid therapy with methylprednisolone sodium succinate (MPSS) after tSCI is a highly debated and controversial topic [2, 7, 10–12]. Specifically, CNS guidelines do not recommend its use [44], whereas AO spine guidelines suggest: (1) “not offering a 24-h infusion of high-dose MPSS to adult patients who present after 8 h with acute SCI”; (2) “a 24-h infusion of high-dose MPSS to adult patients within 8 h of acute SCI as a treatment option” and (3) “not offering a 48-h infusion of high-dose MPSS to adult patients with acute SCI” [45]. We strongly recommend against routine high-dose corticosteroid therapy as proposed in NASCIS I-III studies [10–12]. Some panelists are in favor of the use of corticosteroids at lower doses or in certain patients who may have a favorable risk/benefit ratio. This topic requires further studies, especially in a challenging scenario such as tSCI in polytrauma.

### Notes on the use of the current consensus

This consensus aims to support clinicians’ decision making in the early management of tSCI patients with polytrauma in the first 24 h after injury. The included statements are created to assist a physician’s clinical judgment, which is necessary to provide appropriate (personalized) therapy. Considering the lack of high-quality studies in this setting, we adopted a modified Delphi approach involving experts from different countries worldwide; this approach is less rigorous than evidence-based guidelines. However, our methodology can provide useful and practical recommendations for this challenging clinical scenario. The guidelines promulgated in this work do not represent a standard of practice and have no legal implications. They are suggested plans of care based on the best available evidence and the consensus of experts, but they do not exclude other approaches as being within the standard of practice. Ultimately, responsibility for treatment results rests with those directly engaged therein and not with the consensus group. Moreover, as recommended, we think that a strict collaboration between different medical specialties (i.e., a multidisciplinary approach) is of paramount importance for improving the outcome of these patients. This aspect is of great importance not only in the first 24 h after trauma but also in the long-term care (i.e., high-quality neurorehabilitation).

## Conclusion

In the future, more studies should be encouraged to improve clinical outcomes for polytrauma patients with tSCI. This international multidisciplinary consensus conference was created to provide practical recommendations to deliver the best early possible care for tSCI polytrauma patients in the first 24 h after injury.

### Supplementary Information

**Additional file 1: Appendix 1.**

## Data Availability

The datasets used and/or analyzed during the current study are available from the corresponding author on reasonable request.

## Abbreviations

SCI: Spinal cord injury
tSCI: Traumatic spinal cord injury
WSES: World Society of Emergency Surgery
EANS: European Association of Neurosurgical Societies
TBI: Traumatic brain injury
CT: Computed tomography
MRI: Magnetic resonance imaging
MAP: Mean arterial pressure
Hb: Hemoglobin
RBCT: Red blood cell transfusion
PaO_2_: Arterial partial pressure of oxygen.
PaCO_2_: Arterial partial pressure of carbon dioxide.
PLT: Platelet
PT: Prothrombin time
aPTT: Activated partial thromboplastin time.
POC: Point-of-care
TEG: Thromboelastography
ROTEM: Rotational thromboelastometry
NASCIS: National acute spinal cord injury study
DVT: Deep vein thrombosis
CNS: Congress of neurological surgeons
SCPP: Spinal cord perfusion pressure
ISP: Intraspinal pressure
T1: First thoracic vertebra.
NOAC: Novel oral anticoagulant
PROPPR: Pragmatic randomized optimal platelet and plasma ratios
AAST: American Association for the Surgery of Trauma
ACS-COT: American College of Surgeons Committee on Trauma
DVT: Deep vein thrombosis
MPSS: Methylprednisolone sodium succinate
