# Supplementary material for: Early management of adult traumatic spinal cord injury in patients with polytrauma: a consensus and clinical recommendations jointly developed by the World Society of Emergency Surgery (WSES) & the European Association of Neurosurgical Societies (EANS)
Source: World J Emerg Surg. 2024 Jan 18;19:4. doi: 10.1186/s13017-023-00525-4 (PMC10795357; doi:10.1186/s13017-023-00525-4)
Supplement: Supplementary file 1 — Additional file 1: Appendix 1. [file 13017_2023_525_MOESM1_ESM.docx]

**Appendix 1**

**LIST of PARTICIPANTS to the MULTIDISCIPLINARY CONSENSUS PANEL**

**NEUROSURGERY:**

1. Corrado Iaccarino (Italy)
2. Alessandro Bertuccio (Italy)
3. Andrea Barbanera (Italy)
4. Francesco Costa (Italy)
5. Salman Sharif (Pakistan)
6. Oscar L. Alves (Portugal)
7. Nikolay A. Konovalov (Russia)
8. David O. Okonkwo (USA)
9. Peter J. Hutchinson (UK)
10. Angelos Kolias (UK/Greece)
11. Andreas K. Demetriades (UK/The Netherlands)
12. Deepak Gupta (India)
13. Andres M. Rubiano (Colombia)
14. Alexander R. Vaccaro (USA)
15. Bizhan Aarabi (USA)
16. Michael J. Fehlings (Canada)
17. Peter Gruen (USA)
18. Ilaria Melloni (Italy)
19. Rocco A. Armonda (USA)
20. Ondra Petr (Czech Republic)
21. Andras Buki (Sweden)
22. Ana Maria Castano-Leon (Spain)
23. Peter Toth (Hungary)
24. Sam Klein (Belgium)
25. Niklas Marklund (Sweden)
26. Bart Depreitere (Belgium)
27. Carla Reizinho (Portugal)
28. Ehab Shiban (Germany)
29. Florian Ringel (Germany)
30. Alexander Younsi (Germany)
31. Parmenion P. Tsitsopoulos (Greece)
32. Laura Lippa (Italy)
33. Marcel Ivanov (UK)
34. Teemu M. Luoto (Finland)
35. Andrew I.R. Maas (Belgium)
36. Gregory W.J. Hawriluk (USA)
37. Iftakher Hossain (UK/Finland)
38. Elham Rostami (Sweden)
39. Alfonso Lagares (Spain)
40. Nicole A. Terpolilli (Germany)
41. Marios C. Papadopoulos (UK)
42. Andrzej Maciejczak (Poland)
43. Nikolay Gabrovsky (Bulgaria)
44. Claudius Thomé (Austria)

**ORTHOPEDISTS**

1. Philip F. Stahel (USA)
2. Sergio Mendoza-Lattes (USA)
3. Federico De Iure (Italy)

**ACUTE CARE SURGEONS**

1. Fausto Catena (Italy)
2. Federico Coccolini (Italy)
3. Luca Ansaloni (Italy)
4. Matthew J. Martin (USA)
5. Walter L. Biffl (USA)
6. Raul Coimbra (USA)
7. Fikri M. Abu Zidan (UAE)
8. Miklosh Bala (Israel)
9. Zsolt J. Balogh (Australia)
10. Ronald V. Maier (USA)
11. Kenji Inaba (USA)
12. Sandro Rizoli (Qatar)
13. Andrew W. Kirkpatrick (Canada)
14. Lena M. Napolitano (USA)

Intensivists/neurointensivists/anesthesiologists

1. Lori Shutter (USA)
2. Monica S. Vavilala (USA)
3. Aichholz K. Pudkrong (USA)
4. Edoardo Picetti (Italy)
5. Aarti Sarwal (USA)
6. Chiara Robba (Italy) - non-voting methodologist
7. Rafael Badenes (Spain)
8. Fabio S. Taccone (Belgium)
9. Pierre Bouzat (France)
10. Carlo Coniglio (Italy)
11. Emanuele Russo (Italy)
12. Yasuhiro Otomo (Japan)
13. Monika Zackova (Italy)
14. Daniel Agustin Godoy (Argentina)
15. Geert Meyfroidt (Belgium)
16. Giuseppe Citerio (Italy)
17. Tommaso Zoerle (Italy)
18. Marina Munari (Italy)
19. Frank Rasulo (Italy)
20. Deepak Sharma (USA)
21. Raimund Helbok (Austria)
22. Andrew Udy (Australia)
23. Albert J. Varon (USA)

**NEURORADIOLOGITS**

1. Davide Cerasti (Italy)
2. Enrico Fainardi (Italy)

**EMERGENCY PHYSICIANS**

1. Angelica Loza-Gomez (USA)
2. Zaffer Qasim (USA)

**NEUROLOGISTS**

1. Jan M. Schwab (USA)
